# Supplementary material for: Co-translational binding of importins to nascent proteins
Source: Nat Commun. 2023 Jun 9;14:3418. doi: 10.1038/s41467-023-39150-9 (PMC10256725; doi:10.1038/s41467-023-39150-9)
Supplement: Supplementary file 1 — Supplementary Information [file 41467_2023_39150_MOESM1_ESM.pdf]

Supplementary Information

Co-translational binding of importins to nascent proteins

Authors: Maximilian Seidel, Natalie Romanov, Agnieszka Obarska-Kosinska, Anja Becker, Nayara Trevisan Doimo de Azevedo, Jan Provaznik, Sankarshana R. Nagaraja, Jonathan J. M. Landry, Vladimir Benes and Martin Beck*

Correspondence to: martin.beck@biophys.mpg.de

Supplementary Figures

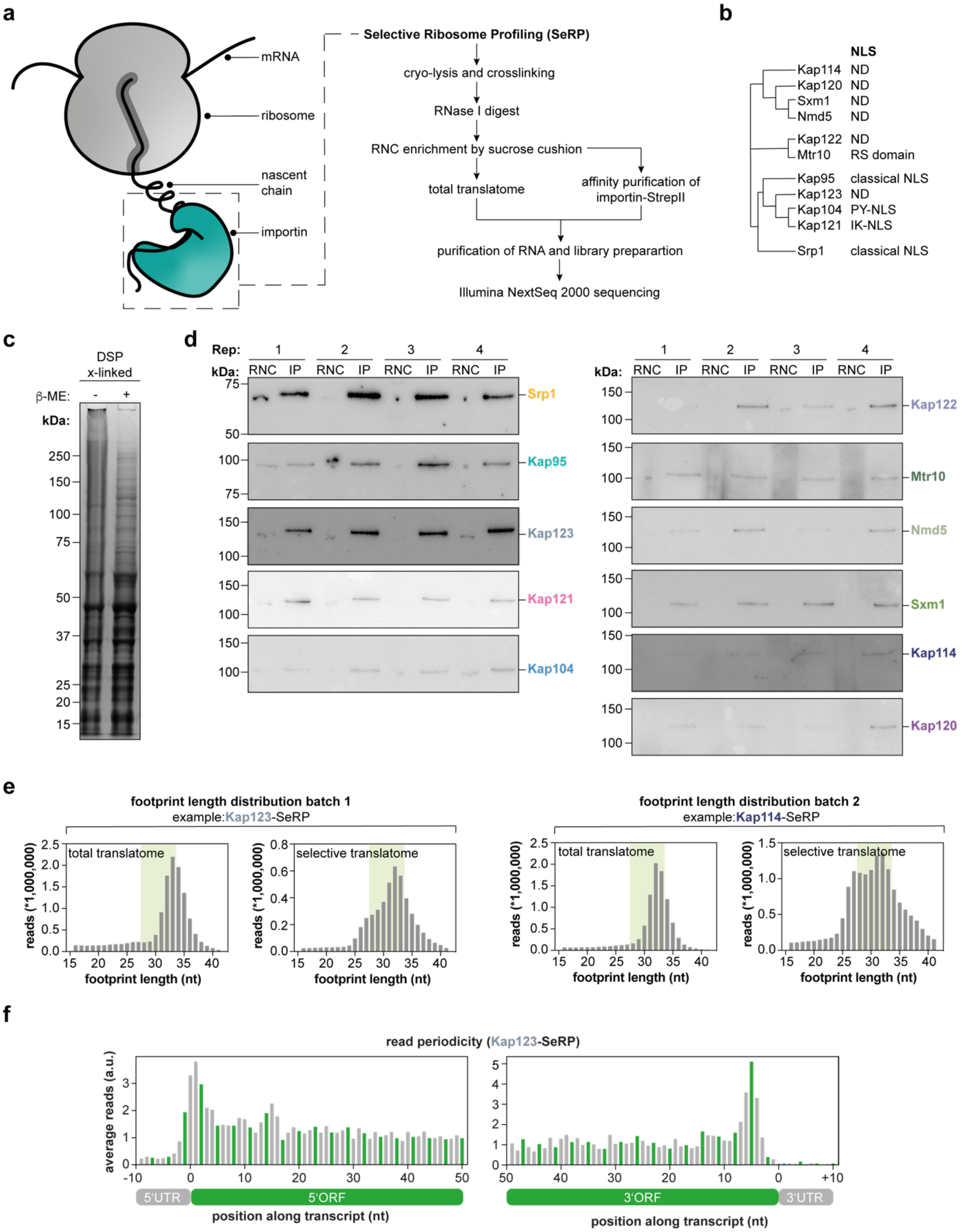

Figure legend on the next page.

Supplementary Figure 1: **a,** Enrichment strategy for importin-bound nascent chains and selective ribosome profiling procedure. **b,** Multiple sequence alignment of importins depicts three distinct groups of importins, consensus sequences in *S. cerevisiae* are indicated if applicable. **c,** Analysis of cross-linking efficiency. To stabilize interactions, lysates were cross-linked in the presence of DSP. Cross-linked lysate shows an upwards shift of protein bands and results in a smear. Reduction of DSP using reducing agents such as β-mercaptoethanol (β-ME) validates that cross-linking was successful under the relevant conditions. Protein was visualized by Coomassie Blue. **d,** Western blot analysis of the individual pulldown experiments. StrepII-tagged importins could be enriched from the ribosome-nascent chain (RNC) fraction. StrepII-tagged protein was detected using an anti-StrepII-tag antibody. **e,** Footprint length distribution of the total translatome and the selective translatome channel. Distribution shows average read distribution across *n*=4 replicates. **f,** Read periodicity plot for the 5' and 3' end of the ORF for the example of Kap123-SeRP of a total translatome. Plots indicate that the footprints align to the ORF and less to the UTRs. Green bars indicate 0-frame and the plot shows an individual replicate. For **c,** and **d,** source data are provided as a Source Data file. ND: not determined; NLS: nuclear localization sequence; x-linked: crosslinked; β-ME: β-mercaptoethanol; Rep: replicate; IP: immunoprecipitation.

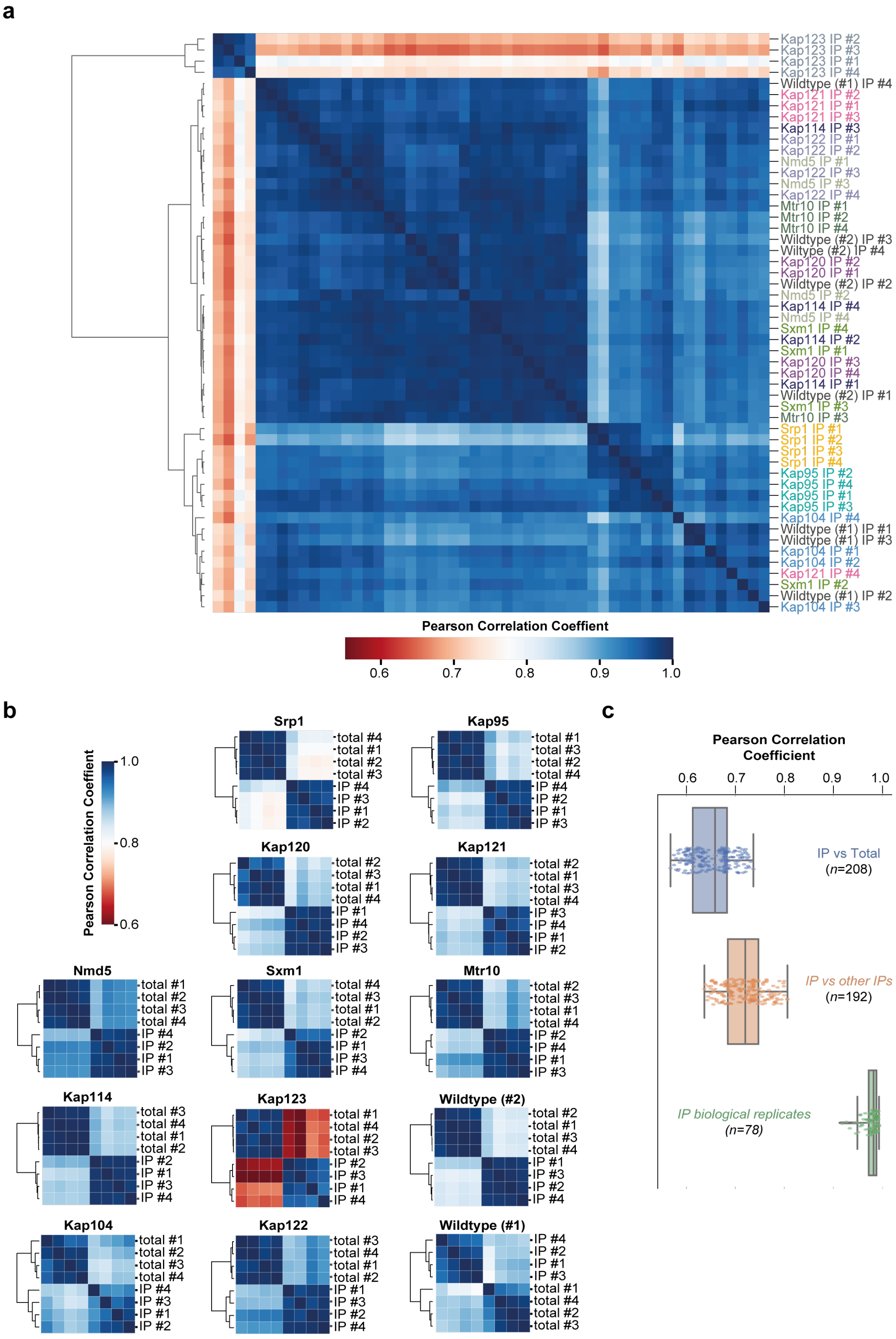

Figure legend on the next page.

Supplementary Figure 2: a, Correlation matrix based on Pearson metric as calculated on all smoothed gene profiles across all replicate IP experiments. Biological replicates are highlighted by color. Hierarchical clustering was conducted using the Euclidean metric, as embedded in the sns.clustermap functionality in Python, **b,** Correlation matrices showing the Pearson correlation values as calculated on all smoothed gene profiles per experiment across total and IP replicates. In all cases, profiles from total replicates cluster distinctly from IP profiles. **c,** Boxplot displaying the Pearson correlation coefficients for indicated combinations, e.g. all combinations of IP vs. total channels (blue), IP vs. other IPs (orange), and correlations within IP biological replicates (green). These values are based on all smoothed profiles of all genes as identified across experiments (the smoothing is applied to avoid issues in sparsity). The central line in the box plots indicates the median, the bottom and top edges of the box the IQR, and the box plot whiskers represent 1.5 times the IQR. Depicted data corresponds to n=4 biologically independent replicates.

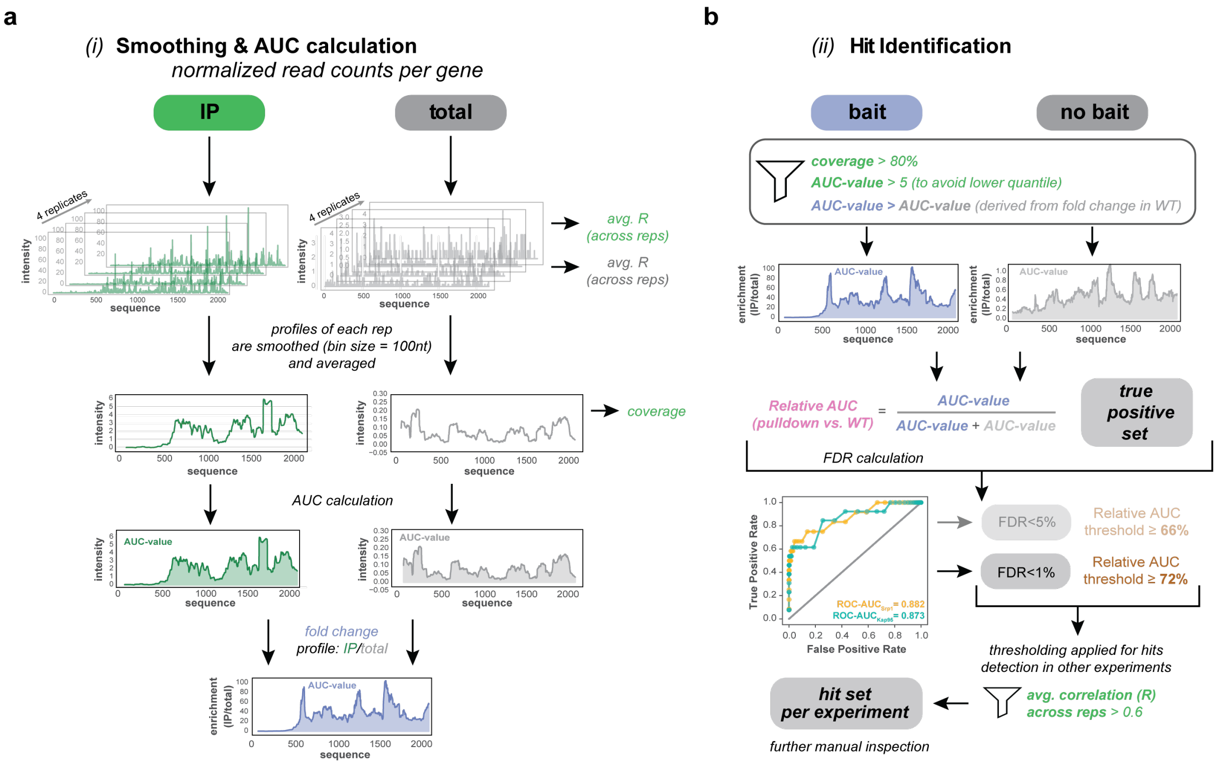

c

SeRP (bait):

	Starting Point	Correlation Filter	Sparsity Filter	AUC Filter	Fold change Filter	FDR Filter 10%	FDR Filter 5%	FDR Filter 1%
Srp1	5452	3259	2843	2842	1416	123	69	17
Kap95	5353	3764	3310	3309	1811	496	220	22
Kap104	5230	1108	848	843	501	30	18	4
Kap114	5458	4087	3618	3614	1893	369	158	21
Kap120	5456	3081	2705	2700	1368	39	26	6
Kap121	5506	3053	2606	2588	1185	14	10	5
Kap122	5328	4076	3527	3526	1911	1284	942	142
Kap123	5412	2434	2083	2079	1203	608	321	31
Mtr10	5443	3098	2707	2702	1412	189	106	19
Sxm1	5411	3183	2761	2757	1544	145	72	12
Nmd5	5442	3895	3418	3417	1834	1519	1192	256

genes remaining: 5000, 4000, 3000, 2000, 1000

Supplementary Figure 3: Hit detection pipeline developed for this study. **a,** Calculation of individual selective ribosome profiles. IP and total translatome (total) were averaged across the four replicates and smoothed with a bin size of 100 nt. Resulting profiles were used to determine the coverage and the area under the curve (AUC)-value in the IP and total, respectively. To obtain a fold change profile or enrichment profile (blue), the IP profile (green) was divided by the associated total profile (grey). **b,** Hit identification across all experiments. The obtained enrichment profiles were filtered according to coverage and AUC-value (see **Materials and Methods** for a more detailed explanation). Each gene-centric enrichment profile is put into context with its corresponding profile in the wildtype condition (no bait), hence a wildtype-relative AUC can be calculated. This relative AUC-value is used for false discovery (FDR) calculation based on the true positive set as defined in **Supplementary Table 1**. A Receiver Operating Curve (ROC) is calculated, indicating the recovery of true hits within the data. At a threshold of 72% (Relative AUC), an FDR of <1% is achieved and hence can be used for filtering highly reliable hits. The final hit set per experiment can be further filtered and manually curated. **c,** Analysis pipeline and the impact of each consecutive filtering step in each of the individual SeRP experiments. Number of remaining genes in each experiment for after each filtering step is indicated.

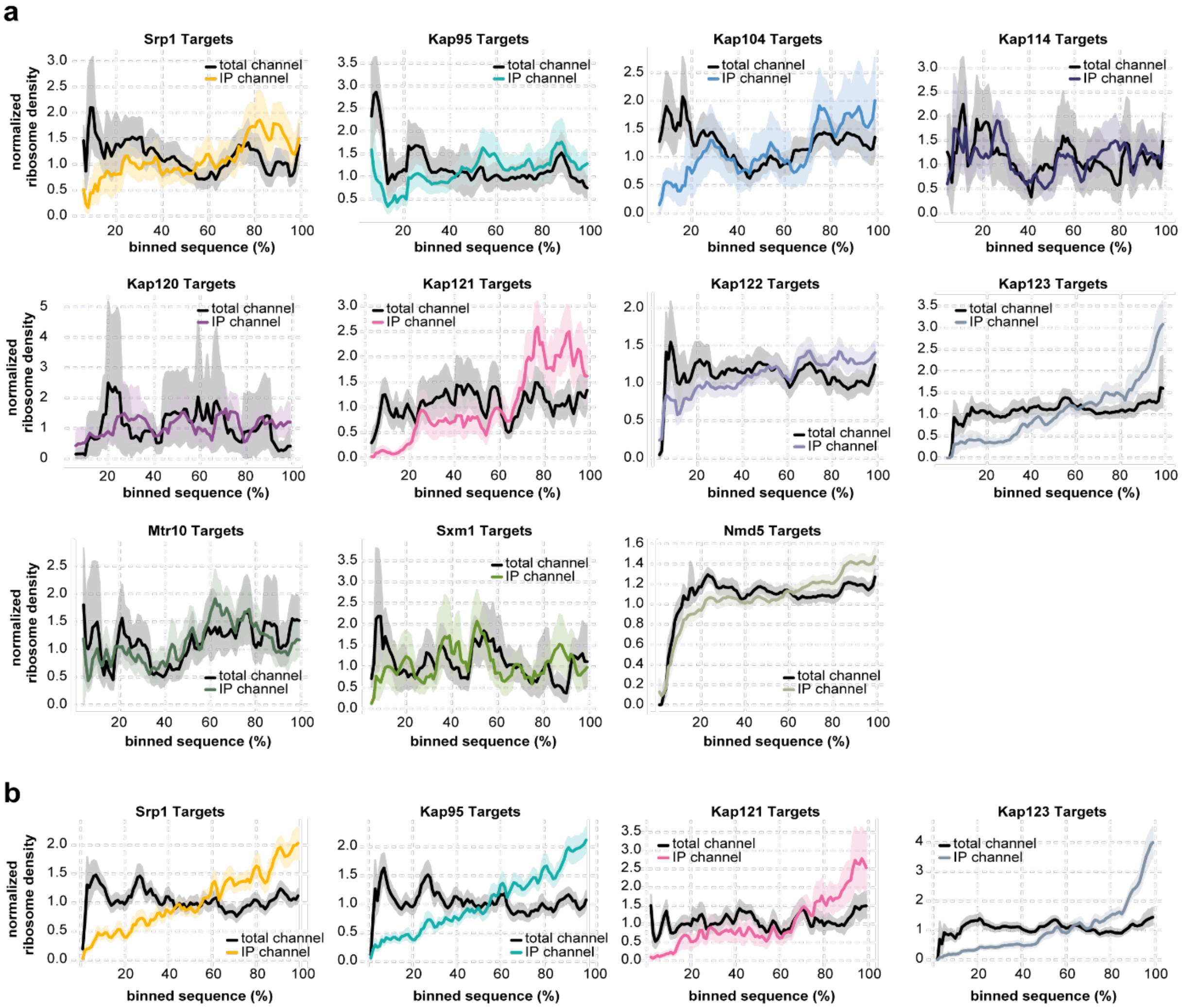

Supplementary Figure 4: Metagene profiles comparing total and IP channels. Metagene plots are shown for targets before (**a**) and after manual inspection (**b**). Total and IP profiles were normalized, respectively, to the sum of normed counts across the profile (with correction for profile length) (y-axis). All profiles across hit genes have been scaled to 100% (x-axis) with aggregating values in respective bins by averaging. The shades signify 95% confidence interval across hits and replicate profiles. Kap123 and Kap121 profiles show a pronounced C-terminal enrichment of footprints in the IP channel.

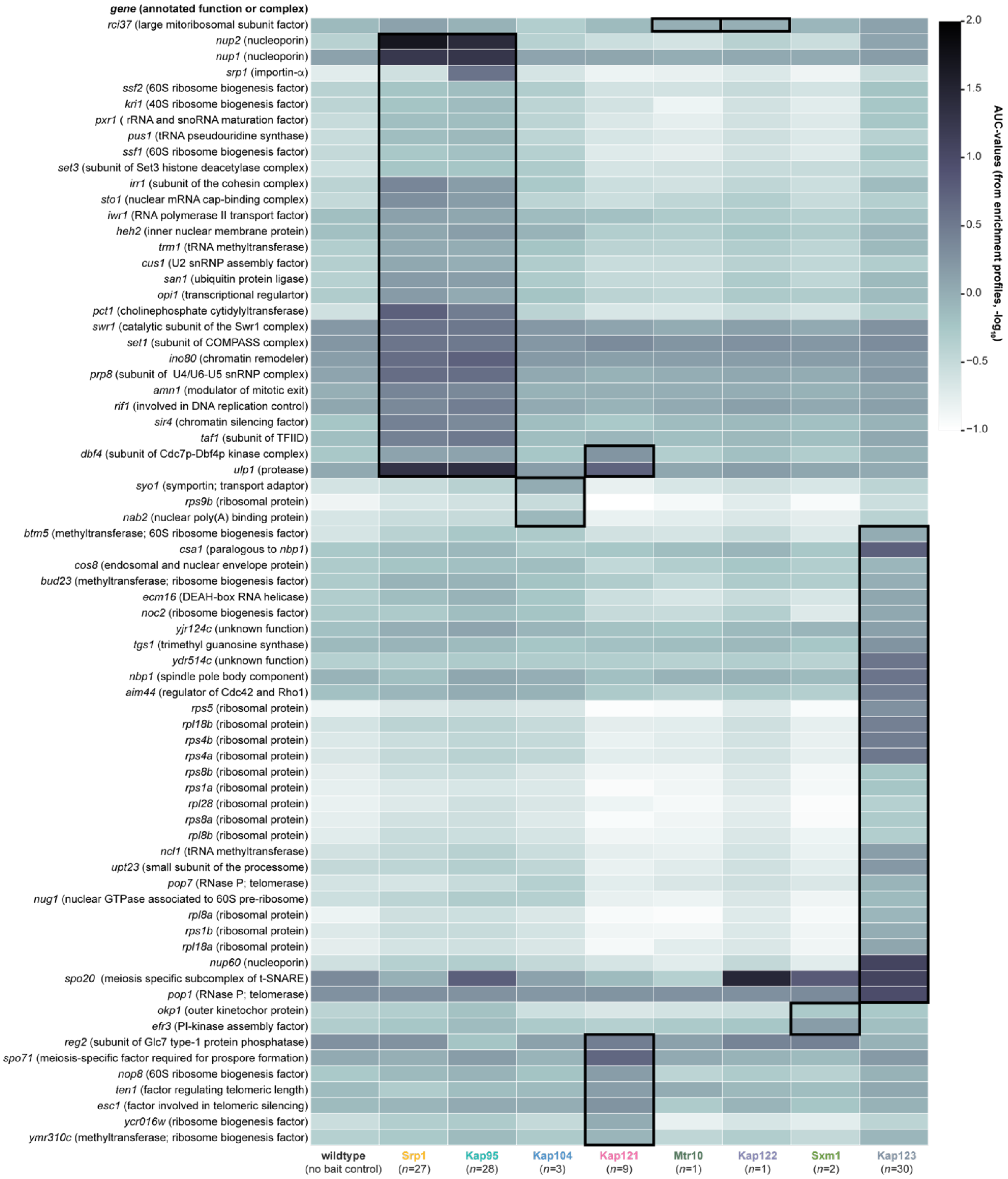

Supplementary Figure 5: AUC-heatmap of all reliably identified hits across different experiments. AUC-values are presented and can be compared to the AUC-values generated in the wildtype (no bait control). Circulated genes represent hits within their respective SeRP experiment.

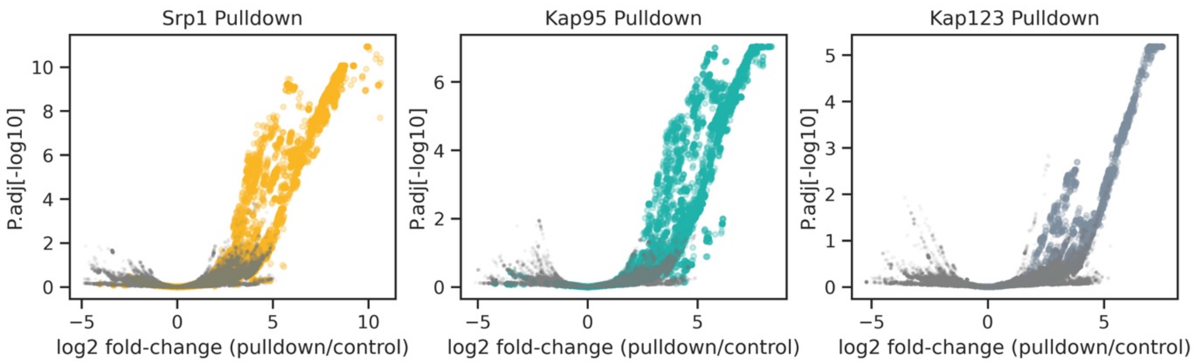

Supplementary Figure 6: Volcano plots illustrating DESeq2-derived results for profiles (normalized counts) across the entire genome for respective pulldown relative to wildtype (no-bait) control (see corresponding **Materials and Methods** section). The x-axis indicates the log2 fold change, and the y-axis the adjusted *P*-value (Benjamini-Hochberg method) in a –log10 scale. Each dot corresponds to a nucleotide position (hence strong cross-correlation in analysis); grey dots signify positions within genes that have not been identified as targets of the respective importin our pipeline, whereas colored dots correspond to genome positions that reside within target or cargo genes. The DESeq2 analysis (based on a negative binomial generalized linear model; two-sided) demonstrates that normalized counts in the identified cargo genes are significantly enriched relative to the no-bait control (Fisher exact test shows $P < 0.0001$ for all three pulldowns). This Supplementary plot shows the underlying data for Srp1, Kap95, and Kap123, where we also found the most reliable hits. The other pulldowns did not demonstrate a significant signal in the DESeq2 analysis.

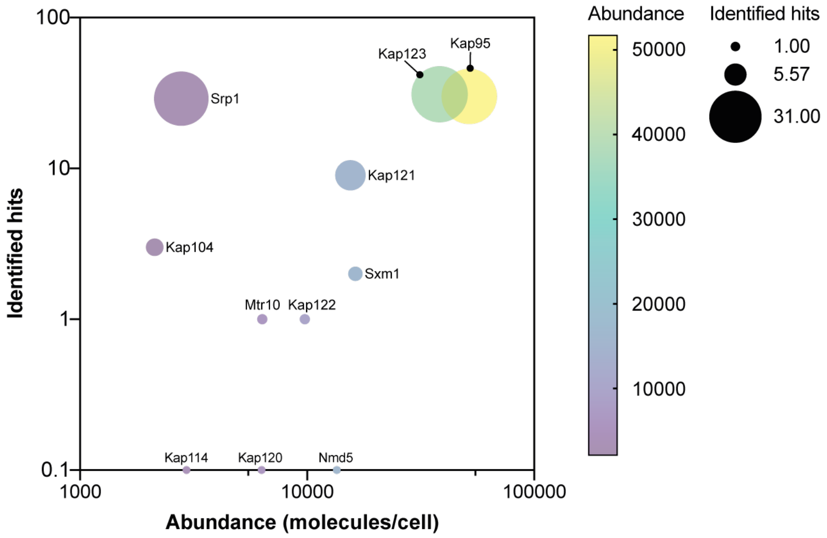

Source: Ghaemmaghami et al, 2003, *Nature.*

Supplementary Figure 7: Relationship of importin abundance and number of identified targets. The analysis reveals that the number of identified hits does not depend on the abundance of the respective importin. Bubble size represents the number of identified hits. Abundance of the respective importin is encoded by a color gradient. Abundances of importins (molecules/cell) were extracted from Ghaemmaghami et al.[1]

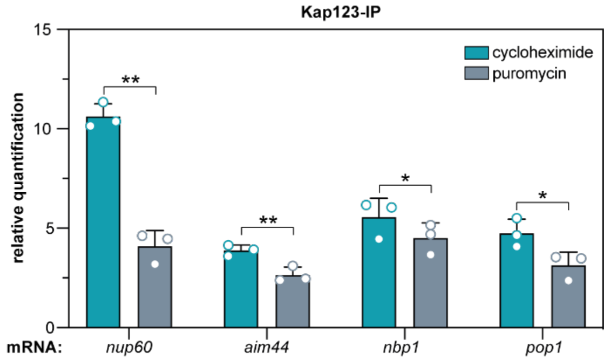

Supplementary Figure 8: RIP-qPCR experiments of a Kap123-StrepII pulldown validates co-translational interactions of Kap123 with nascent cargoes. $n = 3$ biologically independent samples. $^{**}p = 0.0046$ for *nup60*-mRNA; $^{**}p = 0.0098$ for *aim44*-mRNA; $^{*}p = 0.0396$ for *nbp1*-mRNA; *p = 0.0205 for *pop1*-mRNA. Figure depicts mean + SD. ns $p > 0.05$,$^{*}p < 0.05$, $^{**}p < 0.01$ (Two-sided, paired t-test calculated between cycloheximide and puromycin for the respective mRNA). Source data are provided as a Source Data file.

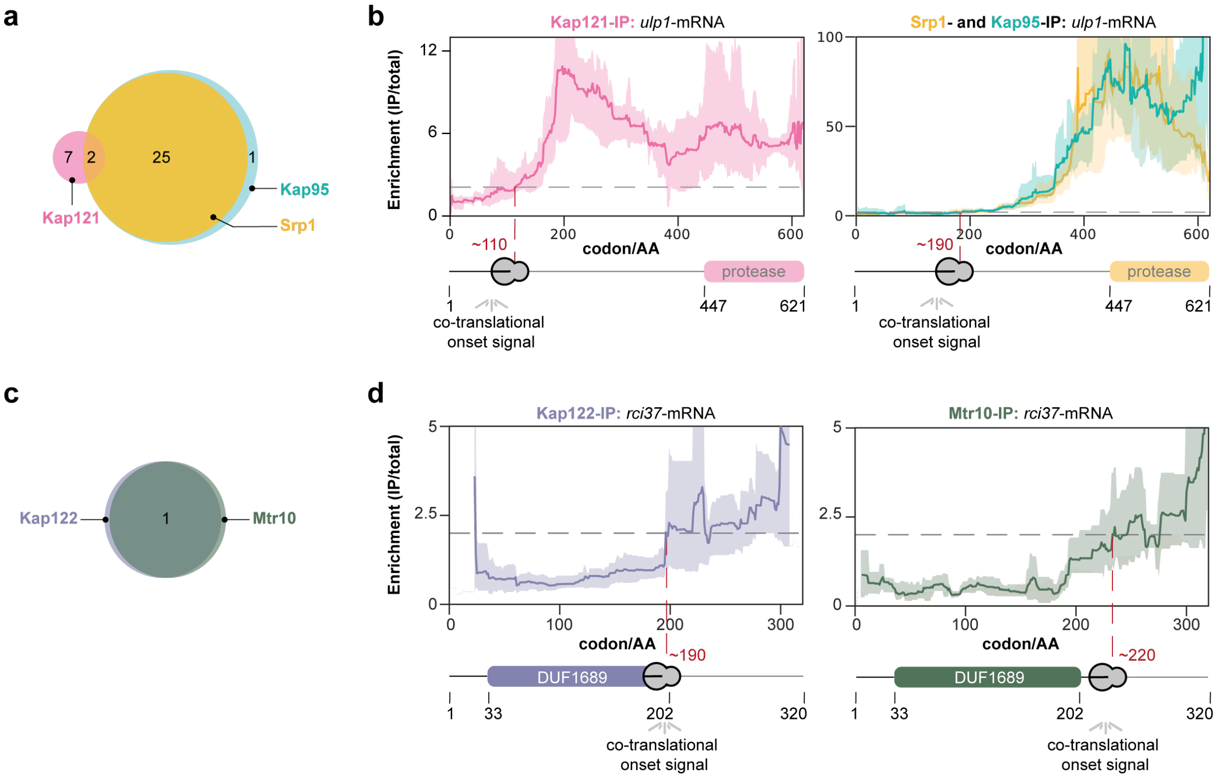

Supplementary Figure 9: Co-translational interactions show minor redundancy. **a,** and **b,** Venn-diagram indicating overlap of the co-translational cargoes between Kap121 and Srp1/Kap95. They share Ulp1 and Dbf4 as substrates but the apparent onset is different implying that Kap121 pioneers in binding to nascent Ulp1. **c,** and **d,** Kap122 and Mtr10 both capture the nascent chain of Rci37. SeRP profiles (IP/total) are shown for the respective mRNA targets from n=4 biologically independent replicates (solid lines are averaged across replicates; shades reflect largest to smalls replicate value interval). Grey dashed lines indicate an arbitrary threshold of 2 used for onset estimation (red dashed line). IP: immunoprecipitation; AA: amino acid; DUF: domain of unknown function.

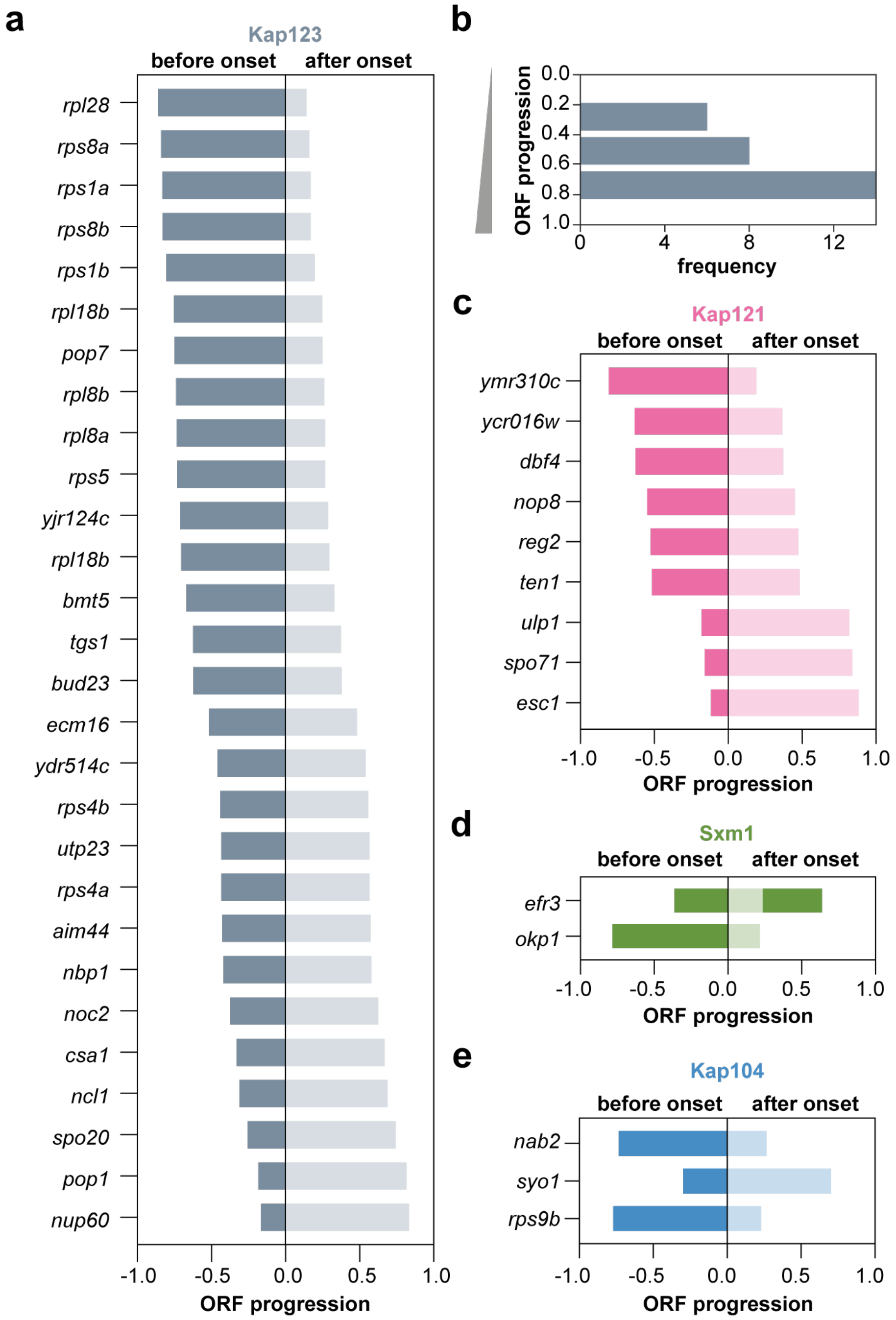

Supplementary Figure 10: Onset distribution across importin-SeRP experiments other than Kap95/Srp1. **a,** Onset distribution for Kap123 cargoes. **b,** The respective histogram indicates a C-terminal bias. Onset plots for **c,** Kap121, **d,** Sxm1, **e,** Kap104. All onset plots show normalized ORF length for all genes. Apparent onsets are centered to zero. ORF: open reading frame.

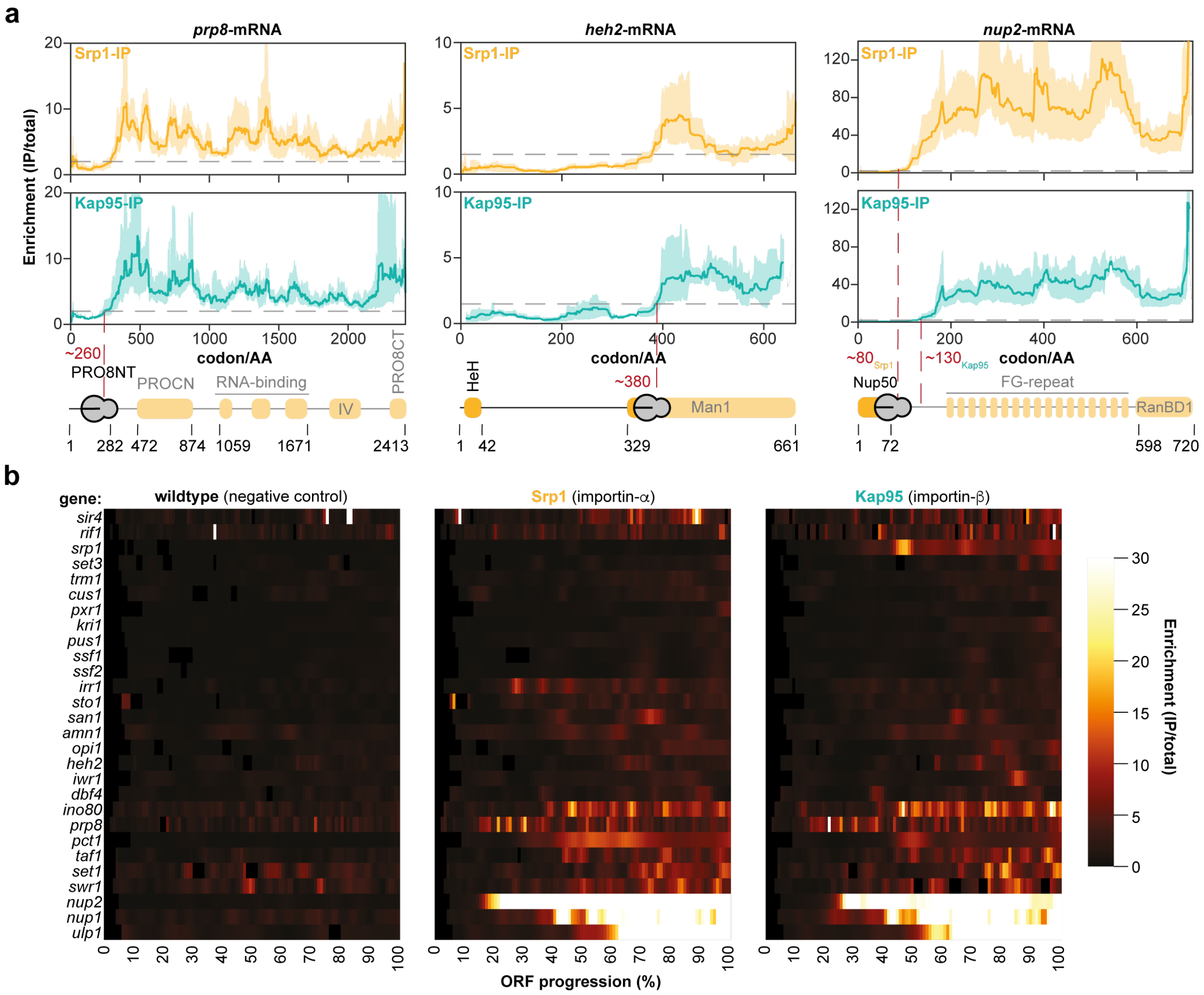

Supplementary Figure 11: Synchronous binding of Srp1 and Kap95 on the nascent chains. **a,** Representative SeRP curves show that Srp1 and Kap95 mutually act on the nascent chains of their cargo in the case of nascent Prp8 and Heh2. In contrast, nascent Nup2 appears to be bound first by Srp1 and subsequently by Kap95. We note that Heh2 expresses an NLS between residues 102-137[2] and requires an additional linker for nuclear localization[3,4]. SeRP of Kap95 and Srp1 suggests binding of importin only upon exposure of the linker from the exit tunnel. SeRP profiles (IP/total) are shown for the respective mRNA targets from n=4 biologically independent replicates (solid lines are averaged across replicates; shades reflect largest to smalls replicate value interval). Grey dashed lines indicate an arbitrary threshold of 2 used for onset estimation (red dashed line). **b,** Heatmap representation of all Srp1 and Kap95 co-translationally targeted cargoes in relation to wildtype (no bait) negative control, showing that signals arise at similar regions within the respective ORFs, while almost no signal is generated in the no-bait control. Heatmap colors indicate the enrichment (IP/total) value according to the scale bar. Each profile and heatmap value were derived from n=4 biologically independent samples for each bait. IP: immunoprecipitation; AA: amino acid; HeH: helix-extension-helix domain; Man1: Man1-Src1p-C-terminal domain; FG-repeat: phenylalanine-glycine repeat; RanBD1: Ran binding domain 1.

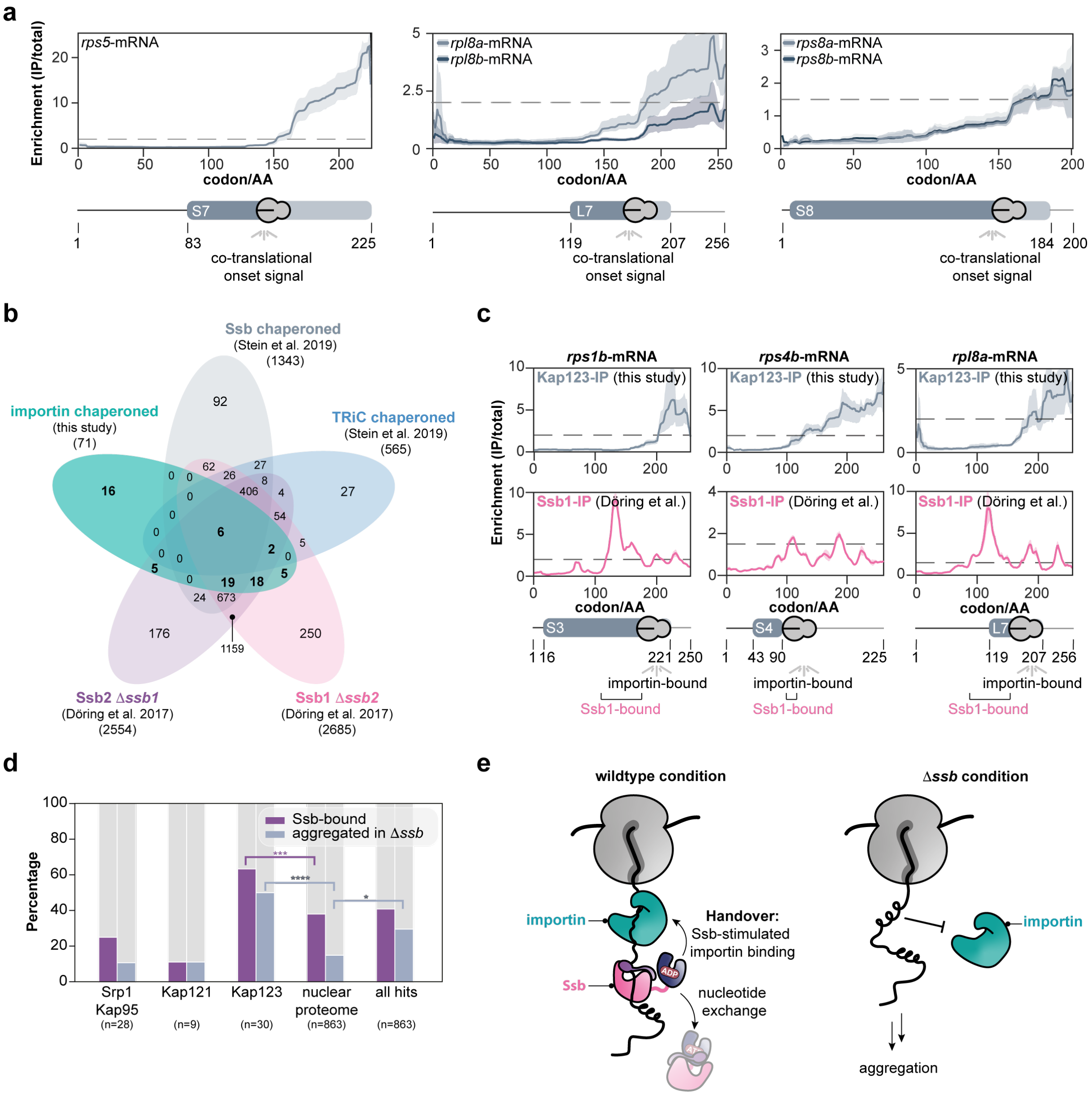

Supplementary Figure 12: Ribosomal proteins captured by Kap123. **a,** Representative SeRP curves for ribosomal proteins. Curves for paralogues ribosomal proteins show similar shapes and onsets but their signal fluctuates according to their expression level. *rpl8a* expression is higher with respect to *rpl8b*, resulting in a stronger signal, while *rps8a* and *rps8b* are expressed at similar levels resulting in two almost identical curves. **b,** Venn-diagram of substrate overlap across Ssb-, TRiC- and importin chaperoned cargo. **c,** Extended evidence that Ssb1 binding to nascent chains precedes Kap123. SeRP profiles (IP/total) are shown for the respective mRNA targets from n=4 biologically independent replicates (this study), Ssb1-SeRP experiments originating from Döring et al.[5] represent profiles generated form n=2 biologically independent replicates (solid lines are averaged across replicates; shades reflect largest to smalls replicate value interval). Grey dashed lines indicate an arbitrary threshold of 1.5 or 2 used for onset estimation (red dashed line). **d,** Kap123 substrates are enriched for proteins that aggregate in the absence of Ssb2. ***P= $7.00\text{x}10^{-03}$ (Kap123 [Ssb-bound], nuclear proteome [Ssb-bound]); ****P=$1.14\text{x}10^{-05}$ (Kap123 [aggregated in Δ*ssb*], nuclear proteome [aggregated in Δ*ssb*]), *P= 0.036 (all hits [aggregated in Δ*ssb*], nuclear proteome [aggregated in Δ*ssb*]). ns $P>$ 0.05, *$P<$ 0.05, **$P<$ 0.01, ***$P<$ 0.005, ****$P<$ 0.001. Fisher Exact test, two-sided. **e,** Possible interpretation: under wildtype conditions, Ssb may maintain importin recognition sites degenerated allowing for importin binding. Within *ssb*Δ, nascent chains undergo folding thus masking the linear NLS and ultimately resulting in aggregation. IP: immunoprecipitation; AA: amino acid.

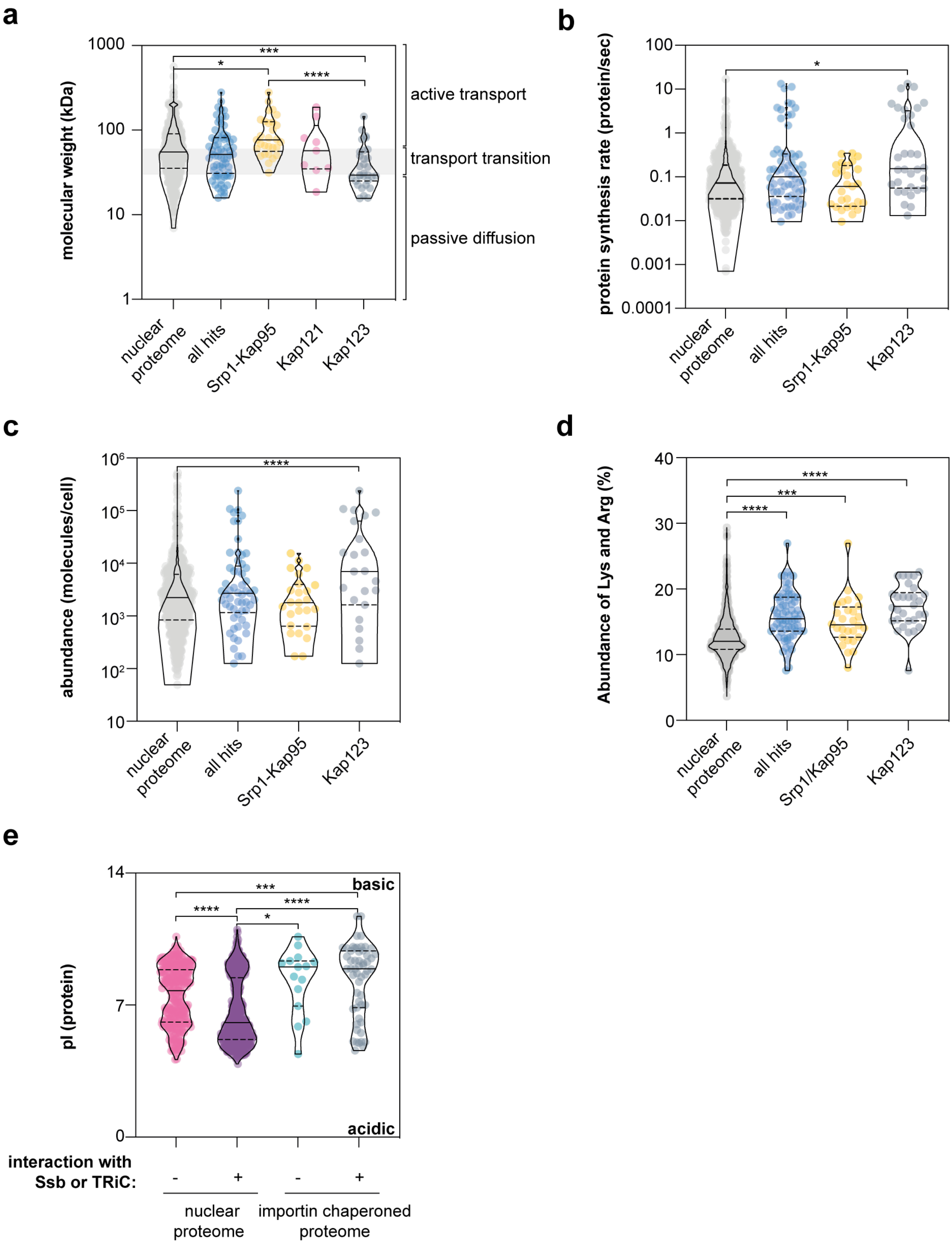

Figure legend on the next page.

Supplementary Figure 13: Extended figures on biophysical parameters that may relate to co-translational chaperoning. **a,** Co-translational chaperoning of cargo is independent from cargo molecular weight. Srp1-Kap95 and Kap121 chaperone cargo that tends to require active transport, while the median size of Kap123 cargo is below the size threshold of permeability barrier for passive diffusion. Figure shows *n*= 857 (nuclear proteome), *n*=71 (all hits), *n*=27 (Srp1-Kap95), *n*=9 (Kap121) and *n*=30 (Kap123) individual proteins. ****P*= 0.0042 (nuclear proteome, Kap123); *****P*=6.41×10^{-5} (Srp1, Kap123), **P*=0.048 (nuclear proteome, Srp1-Kap95). **b,** Kap123 cargoes are proteins with high protein synthesis rate. Figure shows *n*= 774 (nuclear proteome), *n*=69 (all hits), *n*=27 (Srp1-Kap95), and *n*=30 (Kap123) individual proteins. **P*= 0.0227 (nuclear proteome, Kap123). **c,** Kap123 cargoes are highly abundant. Figure shows *n*= 633 (nuclear proteome), *n*=57 (all hits), *n*=25 (Srp1-Kap95), and *n*=23 (Kap123) individual proteins. *****P*=7.17×10^{-4}(nuclear proteome; Kap123). **d,** Proteins bound by importins show higher content of lysine (Lys) and arginine (Arg). "all hits" summarizes all hits across pulldowns. Figure shows *n*= 855 (nuclear proteome), *n*=71 (all hits), *n*=28 (Srp1-Kap95), and *n*=30 (Kap123) individual proteins. *****P*=4.23×10^{-13} (nuclear proteome, all hits); ****P*= 0.0013 (nuclear proteome, Srp1-Kap95); *****P*=3.13×10^{-11} (nuclear proteome, Kap123). **e,** Ssb and TRiC chaperones do not protect proteins with a high pI-value. The plot shows individual proteins: *n*= 327 (nuclear proteome [non-chaperoned]); *n*= 535 (nuclear proteome [Ssb-, TRiC-chaperoned]); *n*= 15 (importin chaperoned proteome [non-chaperoned]); *n*= 56 (importin chaperoned proteome [Ssb-, TRiC-chaperoned]). *****P*= 5.99×10^{-15} (nuclear proteom [non-chaperoned], nuclear proteome [Ssb-, TRiC-chaperoned]); **P*= 0.0122 (nuclear proteome [Ssb-, TRiC-chaperoned]), importin chaperoned proteome [non-chaperoned]); *****P*=6.66×10^{-7} (nuclear proteome [Ssb-, TRiC-chaperoned]), importin chaperoned proteome [Ssb-, TRiC-chaperoned]); ****P*=0.001 (nuclear proteome [non-chaperoned], importin chaperoned proteome [Ssb-, TRiC-chaperoned]). "all hits" summarizes all hits across pulldowns. ns *P*> 0.05, **P*< 0.05, ***P*< 0.01, ****P*< 0.005, *****P*< 0.001. Violin plots show median and quartiles. For all figures, a two-sided Mann-Whitney U-test has been applied to calculate corresponding *P*-values and account for variation in distributions. pI: isoelectric point. Source data are provided as a Source Data file.

Supplementary Tables

Cargo	Importin	Reference	Co-translational interaction detected
Nup1	Kap95, Srp1	shown in [6]	yes
Nup2	Kap95, Srp1	shown in [6,7]	yes
Ulp1	Kap95, Srp1	shown in [8]	yes
Asr1	Kap95	shown in [9]	no
Pct1	Kap95, Srp1	shown in [10]	yes
Heh2	Kap95, Srp1	shown in [4]	yes
Cdc45	Kap95, Srp1	shown in [11]	no
Vps75	Kap95, Srp1	shown in [12]	no
Sla1	Kap95, Srp1	shown in [13]	no
Iwr1	Kap95, Srp1	shown in [14]	yes
Hxk2	Kap95, Srp1	shown in [15]	no
Rrp6	Kap95, Srp1	shown in [16]	no
Siz1	Kap95, Srp1	shown in [17]	no
Upf3	Kap95, Srp1	reviewed in [11]	no
Pcl5	Kap95	shown in [18]	no
Srp1	Kap95	shown in [19]	yes
Sts1	Srp1	shown in [20]	no
Gln3	Srp1	shown in [21]	no
Sto1	Kap95, Srp1	shown in [22,23] and reviewed in [11]	yes
Swi6	Srp1	reviewed in [11]	no
Clb2	Kap95, Srp1	shown in [11]	no
Gcn4	Kap95, Srp1	reviewed in [11]	no
Prp20	Kap95, Srp1	shown in [11]	no
Swi5	Kap95, Srp1	shown in [11]	no
Cdc6	Kap95, Srp1	shown in [11]	no
Prp8	Kap95, Srp1	shown in [24]	yes

Supplementary Table 1: True positive set of cargoes used for the analysis as depicted in **Supplementary Fig.3b**.

Cargo	NLS (residues)	Reference	AF-Score (ipTM+pTM)
Nup1	1-123	[6]	0.65-0.73
Nup2	1-50	[7]	0,76
Ulp1	150-172	[8]	0.78-0,79
Pct1	60-66	[10]	0.64-0.7
Iwr1	9-43	[14]	0.6-0.7
Sto1	2-30	[22,23]	0.8
Prp8	96-117	[24]	0.7
Opi1	109-112	[25]	0.33-0.56
Heh2	124-137	[2]	0.67-0.74
San1	180-200	[26]	0.66-0.76

Supplementary Table 2: Literature reported cNLS for Srp1-Kap95 cargoes. A range of the AF-score is given when the NLS was contained in more than one sequence window. AF: AlphaFold.

Strain Name	Genotype	Source
**BY4741 (wildtype)**	*MATa his3Δ1 leu2Δ0 met15Δ0, ura3Δ0*	provided by Patil lab
**Srp1-StrepII**	(BY4741)*srp1-strepII*	this study
**Kap95-StrepII**	(BY4741)*kap95-strepII*	this study
**Kap104-StrepII**	(BY4741)*kap104-strepII*	this study
**Kap114-StrepII**	(BY4741)*kap114-strepII*	this study
**Kap120-StrepII**	(BY4741)*kap120-strepII*	this study
**Kap121-StrepII**	(BY4741)*kap121-strepII*	this study
**Kap122-StrepII**	(BY4741)*kap122-strepII*	this study
**Kap123-StrepII**	(BY4741)*kap123-strepII*	this study
**Nmd5-StrepII**	(BY4741)*nmd5-strepII*	this study
**Sxm1-StrepII**	(BY4741)*sxm1-strepII*	this study
**Mtr10-StrepII**	(BY4741)*mtr10-strepII*	this study
**GFP**	pRS316 (tef1-promoter::*gfp*::cyc1-terminator::ura3)	this study
**Ino80(NLS)-GFP**	pRS316 (tef1-promoter:*ino80(1255-1374)-gfp*::cyc1-terminator::ura3)	this study
**Prp8(NLS)-GFP**	pRS316 (tef1-promoter::*prp8(502-621)-gfp*::cyc1-terminator::ura3)	this study
**Rps5(NLS)-GFP**	pRS316 (tef1-promoter::*rps5(238-393)-gfp*::cyc1-terminator::ura3)	this study
**Nup60(NLS)-GFP**	pRS316 (tef1-promoter::*nup60(1-159)-gfp*::cyc1-terminator::ura3)	this study
**Pop1(NLS)-GFP**	pRS316 (tef1-promoter::*pop1(316-444)-gfp*::cyc1-terminator::ura3)	this study
**Pct1(NLS-Srp1)-GFP**	pRS316 (tef1-promoter::*pct1(76-195)-gfp*::cyc1-terminator::ura3)	this study
**Pct1(NLS-Kap95)-GFP**	pRS316 (tef1-promoter::*pct1(247-366)-gfp*::cyc1-terminator::ura3)	this study

Supplementary Table 3: Yeast strains and corresponding genotypes used in this study.

Supplementary References

1. Ghaemmaghami, S. *et al.* Global analysis of protein expression in yeast. *Nature* **425**, 737–741 (2003).
2. King, M. C., Lusk, C. P. & Blobel, G. Karyopherin-mediated import of integral inner nuclear membrane proteins. *Nature* **442**, 1003–1007 (2006).
3. Rempel, I. L. *et al.* Flexible and Extended Linker Domains Support Efficient Targeting of Heh2 to the Inner Nuclear Membrane. *Structure* **28**, 185-195.e5 (2020).
4. Meinema, A. C. *et al.* Long unfolded linkers facilitate membrane protein import through the nuclear pore complex. *Science* vol. 333 90–93 (2011).
5. Döring, K. *et al.* Profiling Ssb-Nascent Chain Interactions Reveals Principles of Hsp70-Assisted Folding. *Cell* **170**, 298-311.e20 (2017).
6. Mészáros, N. *et al.* Nuclear Pore Basket Proteins Are Tethered to the Nuclear Envelope and Can Regulate Membrane Curvature. *Dev. Cell* **33**, 285–298 (2015).
7. Matsuura, Y. & Stewart, M. Nup50/Npap60 function in nuclear protein import complex disassembly and importin recycling. *EMBO J.* **24**, 3681–3689 (2005).
8. Panse, V. G., Küster, B., Gerstberger, T. & Hurt, E. Unconventional tethering of Ulp1 to the transport channel of the nuclear pore complex by karyopherins. *Nat. Cell Biol.* **5**, 21–27 (2003).
9. Fries, T. *et al.* A novel conserved nuclear localization signal is recognized by a group of yeast importins. *J. Biol. Chem.* **282**, 19292–19301 (2007).
10. Mackinnon, M. A. *et al.* The Kap60-Kap95 Karyopherin Complex Directly regulates phosphatidylcholine synthesis. *J. Biol. Chem.* **284**, 7376–7384 (2009).
11. Hahn, S., Maurer, P., Caesar, S. & Schlenstedt, G. Classical NLS Proteins from Saccharomyces cerevisiae. *J. Mol. Biol.* **379**, 678–694 (2008).
12. Keck, K. M. & Pemberton, L. F. Interaction with the histone chaperone Vps75 promotes nuclear localization and HAT activity of Rtt109 in vivo. *Traffic* vol. 12 826–839 (2011).
13. Gardiner, F. C., Costa, R. & Ayscough, K. R. Nucleocytoplasmic trafficking is required for functioning of the adaptor protein Sla1p in endocytosis. *Traffic* vol. 8 347–358 (2007).
14. Czeko, E., Seizl, M., Augsberger, C., Mielke, T. & Cramer, P. Iwr1 Directs RNA Polymerase II Nuclear Import. *Mol. Cell* **42**, 261–266 (2011).
15. Peláez, R., Fernández-García, P., Herrero, P. & Moreno, F. Nuclear import of the yeast hexokinase 2 protein requires α/β-importin-dependent pathway. *J. Biol. Chem.* **287**, 3518–3529 (2012).
16. Gonzales-Zubiate, F. A., Okuda, E. K., Da Cunha, J. P. C. & Oliveira, C. C. Identification of karyopherins involved in the nuclear import of RNA exosome subunit Rrp6 in Saccharomyces cerevisiae. *J. Biol. Chem.* **292**, 12267–12284 (2017).
17. Makhnevych, T., Ptak, C., Lusk, C. P., Aitchison, J. D. & Wozniak, R. W. The role of karyopherins in the regulated sumoylation of septins. *J. Cell Biol.* **177**, 39–49 (2007).
18. Streckfuss-Bömeke, K., Schulze, F., Herzog, B., Scholz, E. & Braus, G. H. Degradation of Saccharomyces cerevisiae transcription factor Gcn4 requires a C-terminal nuclear localization signal in the cyclin Pcl5. *Eukaryot. Cell* **8**, 496–510 (2009).
19. Cingolani, G., Petosa, C., Weis, K. & Müller, C. W. Structure of importin-β bound to the IBB domain of importin-α. *Nature* **399**, 221–229 (1999).
20. Ha, S. W., Ju, D. & Xie, Y. Nuclear import factor Srp1 and its associated protein Sts1 couple ribosome-bound nascent polypeptides to proteasomes for cotranslational degradation. *J. Biol. Chem.* **289**, 2701–2710 (2014).
21. Carvalho, J., Bertram, P. G., Wente, S. R. & Zheng, X. F. S. Phosphorylation Regulates the Interaction between Gln3p and the Nuclear Import Factor Srp1p. *J. Biol. Chem.* **276**, 25359–25365 (2001).

22. Marfori, M., Lonhienne, T. G., Forwood, J. K. & Kobe, B. Structural Basis of High-Affinity Nuclear Localization Signal Interactions with Importin-α. *Traffic* **13**, 532–548 (2012).
23. Dias, S. M. G., Wilson, K. F., Rojas, K. S., Ambrosio, A. L. B. & Cerione, R. A. The molecular basis for the regulation of the cap-binding complex by the importins. *Nat. Struct. Mol. Biol.* **16**, 930–937 (2009).
24. Boon, K. L. *et al.* Prp8 mutations that cause human retinitis pigmentosa lead to a U5 snRNP maturation defect in yeast. *Nat. Struct. Mol. Biol.* **14**, 1077–1083 (2007).
25. Hofbauer, H. F. *et al.* The molecular recognition of phosphatidic acid by an amphipathic helix in Opi1. *J. Cell Biol.* **217**, 3109–3126 (2018).
26. Fredrickson, E. K., Candadai, S. V. C., Tam, C. H. & Gardner, R. G. Means of self-preservation: How an intrinsically disordered ubiquitin-protein ligase averts self-destruction. *Mol. Biol. Cell* **24**, 1041–1052 (2013).
