# Supplementary material for: Co-translational binding of importins to nascent proteins
Source: Nat Commun. 2023 Jun 9;14:3418. doi: 10.1038/s41467-023-39150-9 (PMC10256725; doi:10.1038/s41467-023-39150-9)
Supplement: Supplementary file 3 — Description of Additional Supplementary Files' [file 41467_2023_39150_MOESM3_ESM.docx]

Physical parameters and scores of protein-protein interfaces modeled by AlphaFold-Multimer. The columns show the following: “Model” – name of the target and region (in AA) that was modeled, “Interface residues” – number of residues at the interface, “Polar”, ”Hydrophobic”, and “Charged” – fractions of the corresponding type of residues at the interface, “Hydrogen bonds” – number of potential hydrogen bonds, “Salt bridges” – number of potential salt bridges, “Solvation free energy” - solvation free energy gain upon formation of the interface, “P-value” - the P-value of the observed solvation free energy gain (P-value < 0.5 implies higher likelihood that the interaction is specific^1^,“PI score” – PI score of the interface, with values > 0 indicating that the interface resembles real interfaces. All values were calculated with PI_score pipeline^2^ using CCP4^3^ and PISA^1^ programs included in the pipeline. The models and crystal structures were minimized using GROMACS^4^ prior to analysis. Cells with ipTM+pTM score color by value, from high to low, using color gradient from red to green. Blank cells mean that no interface was detected by PI_score.

References:

1. Krissinel, E. & Henrick, K. Inference of Macromolecular Assemblies from Crystalline State. *J. Mol. Biol.* **372**, 774–797 (2007).

2. Malhotra, S., Joseph, A. P., Thiyagalingam, J. & Topf, M. Assessment of protein–protein interfaces in cryo-EM derived assemblies. *Nat. Commun.* **12**, (2021).

3. Winn, M. D. *et al.* Overview of the CCP4 suite and current developments. *Acta Crystallogr. Sect. D Biol. Crystallogr.* **67**, 235–242 (2011).

4. Abraham, M. J. *et al.* Gromacs: High performance molecular simulations through multi-level parallelism from laptops to supercomputers. *SoftwareX* **1**–**2**, 19–25 (2015).
