# Supplementary material for: Co-translational binding of importins to nascent proteins
Source: Nat Commun. 2023 Jun 9;14:3418. doi: 10.1038/s41467-023-39150-9 (PMC10256725; doi:10.1038/s41467-023-39150-9)
Supplement: Supplementary file 5 — Reporting Summary [file 41467_2023_39150_MOESM5_ESM.pdf]

nature research

Corresponding author(s): Martin Beck

Last updated by author(s): May 23, 2023

Reporting Summary

Nature Research wishes to improve the reproducibility of the work that we publish. This form provides structure for consistency and transparency in reporting. For further information on Nature Research policies, see Authors & Referees and the Editorial Policy Checklist .

Statistics

For all statistical analyses, confirm that the following items are present in the figure legend, table legend, main text, or Methods section.

n/a	Confirmed	
☐	☒	The exact sample size (*n*) for each experimental group/condition, given as a discrete number and unit of measurement
☐	☒	A statement on whether measurements were taken from distinct samples or whether the same sample was measured repeatedly
☐	☒	The statistical test(s) used AND whether they are one- or two-sided<br>*Only common tests should be described solely by name; describe more complex techniques in the Methods section.*
☐	☒	A description of all covariates tested
☐	☒	A description of any assumptions or corrections, such as tests of normality and adjustment for multiple comparisons
☐	☒	A full description of the statistical parameters including central tendency (e.g. means) or other basic estimates (e.g. regression coefficient) AND variation (e.g. standard deviation) or associated estimates of uncertainty (e.g. confidence intervals)
☐	☒	For null hypothesis testing, the test statistic (e.g. *F*, *t*, *r*) with confidence intervals, effect sizes, degrees of freedom and *P* value noted<br>*Give P values as exact values whenever suitable.*
☐	☒	For Bayesian analysis, information on the choice of priors and Markov chain Monte Carlo settings
☐	☒	For hierarchical and complex designs, identification of the appropriate level for tests and full reporting of outcomes
☐	☒	Estimates of effect sizes (e.g. Cohen's *d*, Pearson's *r*), indicating how they were calculated

Our web collection on statistics for biologists contains articles on many of the points above.

Software and code

Policy information about availability of computer code

Data collection	Images were collected using the Leica LAS X software (v.4.4.0.24861).

Data analysis	Used software are described in the Material and Methods section:<br>GraphPad Prism (v9.0.0) is commercially available at https://www.graphpad.com/scientific-software/prism/. MATLAB (v9.7.0.1296695 (R2019b) Update 4) is commercially available under https://www.mathworks.com/downloads/. tophat2 (v2.0.10) can be downloaded at https://ccb.jhu.edu/software/tophat/index.shtml. cutadapt (v2.3) (Martin, 2011) can be installed at https://cutadapt.readthedocs.io/en/v2.3/installation.html. A significant part of the data analysis has been conducted in Python (v3.6.12) applying the numpy (v1.19.2), scipy (v1.5.2) and pandas (v.1.1.5) package, and for visualization purposes the matplotlib (v3.3.2) and seaborn (v0.11.1) package.<br>For initial SeRP data analysis, we used previously published scripts https://doi.org/10.5281/zenodo.2602493.<br>For AlphaFold modeling, following softwares were requried: AlphaFold (v.2.2.0) (https://github.com/deepmind/alphafold/releases/tag/v2.2.0), ccp4 (https://www.ccp4.ac.uk/download/#os=mac), PI-score (https://gitlab.com/topf-lab/pi_score) and JalView (v 2.11.2.3) (http://www.jalview.org/getdown/release/). Structures were analyzed using publically avaiable UCSF Chimera (v1.5) that can be downloaded at https://www.cgl.ucsf.edu/chimera/download.html and ChimeraX (v.1.3.dev202110160902 (2021-10-16)) which is accessible under https://www.rbvi.ucsf.edu/chimerax.<br>Images were analyzed using ImageJ (v.1.52), which can be downloaded at https://imagej.nih.gov/ij/download.html.<br>qPCR was analyzed using QuantStudio (v1.5.1) which is open access and can be downloaded at https://www.thermofisher.com/de/de/home/global/forms/life-science/quantstudio-3-5-software.html.<br>pI-values were determined using expasy (https://web.expasy.org/compute_pi/). Domains for domain-onset relationship was determined using InterPro (release 89.0).<br>The ribosome profiling processing pipeline can be obtained from Zenodo (DOI: 10.5281/zenodo.2602493). Our previously published MatLab scripts are deposited in Zenodo (DOI: 10.5281/zenodo.5887402). The updated version and the SeRP hit identification pipeline is available on Zenodo (DOI: 10.5281/zenodo.7753270). AlphaFold modeling of Srp1 bound to NLS was carried out using AlphaPulldown (https://github.com/KosinskiLab/AlphaPulldown).

For manuscripts utilizing custom algorithms or software that are central to the research but not yet described in published literature, software must be made available to editors/reviewers. We strongly encourage code deposition in a community repository (e.g. GitHub). See the Nature Research guidelines for submitting code & software for further information.

Data

Policy information about availability of data

All manuscripts must include a data availability statement. This statement should provide the following information, where applicable:

- Accession codes, unique identifiers, or web links for publicly available datasets
- A list of figures that have associated raw data
- A description of any restrictions on data availability

previously published data (metadata), and data of Ssb1/217 and TRiC16 is available in a Source Data file.
The selective ribosome profiling data for all experiments conducted in this study are available on the European Nucleotide Archive database under the accession code PRJEB53855 [https://www.ebi.ac.uk/ena/browser/view/PRJEB53855]. For the initial processing of the ribosome-protected footprints, we used a script suite for selective ribosome profiling.14 Furthermore, we provide script on Zenodo [https://doi.org/10.5281/zenodo.2602493] and [https://doi.org/10.5281/zenodo.7753270], which can be used to process ribosome profiling data and to generate selective ribosome profiles. Note, all selective ribosome profiles for the entire coding genome of S. cerevisiae is available on Zenodo [https://doi.org/10.5281/zenodo.7753270]. This script suite also provides the required reference genome files for Saccharomyces cerevisiae (S288C_reference_sequence_R64-1-1_20110203.fsa), as downloaded from SGD [http://sgd-archive.yeastgenome.org/sequence/S288C_reference/genome_releases/S288C_reference_genome_R64-1-1_20110203.tgz]. We also used sacCer3.ensgene.gtf for mapping introns and exons, as extracted from Ensembl 70 [https://hgdownload.soe.ucsc.edu/goldenPath/sacCer3/bigZips/genes/sacCer3.ensGene.gtf.gz].
Previously published SeRP data for Ssb1/217 and Ssb and TRiC16 can be accessed at Gene Expression Omnibus (GEO) under GEO: GSE93830 [https://www.ncbi.nlm.nih.gov/geo/query/acc.cgi?acc=GSE93830] and GEO: GSE114882 [https://www.ncbi.nlm.nih.gov/geo/query/acc.cgi?acc=GSE114882].
The structure of the autoinhibitory NLS of Srp1 bound to Srp1 (Fig. 3a) can be found in the Protein Data Bank under the accession number PDB: 1WA5 [10.2210/pdb1WA5/pdb].39 The ribosome structure (Fig. 4) can be accessed under PDB: [10.2210/pdb4V7R/pdb]46. AlphaFold-Multimer models will be made available upon publication and can be requested by the reviewers. AlphaFold models of the r-proteins (Fig. 5) originate from the AlphaFold database (https://alphafold.ebi.ac.uk/).48,49 Structures can be accessed under [https://alphafold.ebi.ac.uk/entry/A0A0J9XHQ9] (Rps5), [https://alphafold.ebi.ac.uk/entry/A0A1X7R1F4] (Rpl18a), and https://alphafold.ebi.ac.uk/entry/A0A0F7RSH3 (Rpl28).

Field-specific reporting

Please select the one below that is the best fit for your research. If you are not sure, read the appropriate sections before making your selection.

[x] Life sciences [ ] Behavioural & social sciences [ ] Ecological, evolutionary & environmental sciences

For a reference copy of the document with all sections, see nature.com/documents/nr-reporting-summary-flat.pdf

Life sciences study design

All studies must disclose on these points even when the disclosure is negative.

Sample size	We did not perform sample size calculations and conducted our experiments in analogy to the previously published studies by Shiber et al. (2018) and Seidel et al. (2022).
Data exclusions	No data was excluded from the analysis.

Replication	A minimum of two independent biological replicates was conducted for imaging experiments. All SeRP experiments were conducted with four biologically independent replicates. Exact numbers of replicates are indicated in figure legends.
Randomization	We did not use randomization as the study did not include large datasets.
Blinding	Blinding was not required for the data that we present here.

Reporting for specific materials, systems and methods

We require information from authors about some types of materials, experimental systems and methods used in many studies. Here, indicate whether each material, system or method listed is relevant to your study. If you are not sure if a list item applies to your research, read the appropriate section before selecting a response.

Materials & experimental systems

n/a	Involved in the study
☐	☒ Antibodies
☐	☒ Eukaryotic cell lines
☒	☐ Palaeontology
☒	☐ Animals and other organisms
☒	☐ Human research participants
☒	☐ Clinical data

Methods

n/a	Involved in the study
☒	☐ ChIP-seq
☒	☐ Flow cytometry
☒	☐ MRI-based neuroimaging

Antibodies

Antibodies used	Monoclonal antibody against StrepII-tag (1:1,000) was purchased from abcam (EPR12666; ab180957; Lot. No. GR3212622-7) and anti-rabbit IgG, HRP-linked antibody (1:10,000) was purchased from Jackson ImmunoResearch (RRID AB_2313567).
Validation	The antibodies used in this study were previously validated in other studies (compare to manufacture's references). We did not detect any signal on membranes of wildtype BY4741 RNC pellets or mock IPs underscoring the specificity of the chemiluminescent signal (negative wildtype control is shown in Source Data). According to the manufacturer, the anti-StepII antibody is species independent and have been shown to react with StrepII-tagged proteins (positive control).

Eukaryotic cell lines

Policy information about cell lines

Cell line source(s)

Strains (Mating_type/ Strain_Name/ Genotype/ Created
1) alpha/ BY4741/ MATa his31 leu20 met150, ura30 / provides by Patil Lab
2) alpha/ Srp1-StrepII/ MATa his31 leu20 met150, ura30 srp1-strepII / this study
3) alpha/ Kap95-StrepII/ MATa his31 leu20 met150, ura30 kap95-strepII / this study
4) alpha/ Kap104-StrepII/ MATa his31 leu20 met150, ura30 kap104-strepII / this study
5) alpha/ Kap114-StrepII/ MATa his31 leu20 met150, ura30 kap114-strepII / this study
6) alpha/ Kap120-StrepII/ MATa his31 leu20 met150, ura30 kap120-strepII / this study
7) alpha/ Kap121-StrepII/ MATa his31 leu20 met150, ura30 kap121-strepII / this study
8) alpha/ Kap122-StrepII/ MATa his31 leu20 met150, ura30 kap122-strepII / this study
9) alpha/ Kap123-StrepII/ MATa his31 leu20 met150, ura30 kap123-strepII / this study
10) alpha/ Nmd5-StrepII/ MATa his31 leu20 met150, ura30 nmd5-strepII / this study
11) alpha/ Sxm1-StrepII/ MATa his31 leu20 met150, ura30 sxm1-strepII / this study
12) alpha/ Mtr10-StrepII/ MATa his31 leu20 met150, ura30 mtr10-strepII / this study
12) alpha/ GFP/ MATa his31 leu20 met150, ura30 pRS316(tef1-promoter::gfp::cyc1-terminator::ura3/ this study
13) alpha/ Ino80(NLS)-GFP/ MATa his31 leu20 met150, ura30 pRS316(tef1-promoter::ino80(1255-1374)-gfp::cyc1-terminator::ura3/ this study
14) alpha/ Prp8(NLS)-GFP/ MATa his31 leu20 met150, ura30 pRS316(tef1-promoter::prp8(502-621)-gfp::cyc1-terminator::ura3/ this study
15) alpha/ Rps5(NLS)-GFP/ MATa his31 leu20 met150, ura30 pRS316(tef1-promoter::rps5(238-393)-gfp::cyc1-terminator::ura3/ this study
16) alpha/ Nup60(NLS)-GFP/ MATa his31 leu20 met150, ura30 pRS316(tef1-promoter::nup60(1-159)-gfp::cyc1-terminator::ura3/ this study
17) alpha/ Pop1(NLS)-GFP/ MATa his31 leu20 met150, ura30 pRS316(tef1-promoter::pop1(316-444)-gfp::cyc1-terminator::ura3/ this study
18) alpha/ Pct1(NLS-Srp1)-GFP / MATa his31 leu20 met150, ura30 pRS316(tef1-promoter::pct1(76-195)-gfp::cyc1-terminator::ura3/ this study
19) alpha/ Pct1(NLS-Kap95)-GFP/ MATa his31 leu20 met150, ura30 pRS316(tef1-promoter::pct1(247-366)-gfp::cyc1-terminator::ura3/ this study

Authentication	n.a.
Mycoplasma contamination	No test was needed since only S. cerevisiae strains were used
Commonly misidentified lines (See ICLAC register)	No commonly misidentified cell lines were used
